# Determining capacity of people with dementia to take part in research: an electronic survey study of researcher confidence, competence and training needs

**DOI:** 10.1186/s12910-024-01056-6

**Published:** 2024-05-28

**Authors:** Sarah Griffiths, Victoria Shepherd, Anna Volkmer

**Affiliations:** 1 Centre for Ageing Population Studies, Primary Care and Population Health, University College London, London, UK; 2 Centre for Trials Research, Cardiff University, Cardiff, UK; 3 Psychology and Language Sciences, University College London, London, UK

**Keywords:** Capacity, Consent, Dementia, Decision-making, Supported decision-making

## Abstract

**Background:**

Researchers are required to determine whether a person has capacity to consent to a research study before they are able to participate. The Mental Capacity Act and accompanying Code of Practice for England and Wales provide some guidance on this process, but researchers have identified that it can be difficult to determine capacity to consent when a person has complex cognitive or communication needs. This study aimed to understand the experiences and opinions of researchers who recruit people with dementia to research projects, to inform the future development of training resources.

**Methods:**

A mixed method, cross-sectional, electronic survey was circulated via social media and research networks in England and Wales. The survey remained open for ten weeks and included open and closed questions exploring respondents’ confidence in determining capacity in the context of recruiting people with dementia to consent, their views on training and support they have experienced and their suggestions for future training and support needs.

**Results:**

60 respondents completed the survey from across England and Wales. Although 75% of respondents had experience of determining capacity to consent with people with dementia to research, only 13% rated themselves as feeling ‘very confident’ in this. Qualitative content analysis of open responses led to the generation of six themes, explaining researchers’ confidence, competence and future training needs in this area: (1) Researcher uncertainties, (2) Lack of time, (3) Balancing information complexity with accessibility, (4) Gatekeepers, (5) Existing enablers and (6) Envisioning future training.

**Conclusions:**

Researchers would benefit from specific training in undertaking conversations around consent with people with dementia. People with dementia may have fluctuating capacity, and despite support from caregivers, researchers have little practical guidance on methods of determining a person’s ability to understand or appreciate the information they have provided during the consent process. Given the development of large complex trials within dementia research, there is an urgency to develop specific and practical guidance and training for researchers working with people with dementia and their families.

**Supplementary Information:**

The online version contains supplementary material available at 10.1186/s12910-024-01056-6.

## Background

Individuals with conditions affecting capacity are an under-represented population in research [1]. Inequity in consent processes likely contributes to this disparity; people with capacity affecting conditions are often assumed to be unable to make consent decisions and therefore routinely excluded from social, clinical and biomedical research [2]. Consequently, the evidence-base for care and support for populations who may lack mental capacity is inferior compared to other populations. This, in turn, contributes to societal healthcare inequalities, whereby people with dementia are less likely than other more researched groups to be able to access the care and support they need [3]. Conversely, including people with dementia, who may or may not lack capacity, in research will ensure that health and social care providers can understand and meet these needs. There is, therefore, an urgent need to address barriers to research participation for people who may lack mental capacity.

People with dementia are one such population who may lack the ability to consent [4] and are frequently excluded from research studies [5]. The Mental Capacity Act (MCA) [6] outlines the process to follow in England and Wales if an adult lacks capacity to make a certain decision at a specific time, due to an impairment or disturbance in the functioning of the brain or mind. Sections 30–34 of the MCA provide broad guidelines for conducting research with adults who lack capacity. Although clinical trials are governed under Clinical Trials Regulations [7] (schedule 1, Part 5) the MCA remains applicable to determining capacity of trial participants. The Mental Capacity Act 2005: Code of Practice [8] explains that ‘*Researchers should assume that a person has capacity, unless there is proof that they lack capacity to make a specific decision. The person must also receive support to try to help them make their own decision*’ (p204). The Code of Practice provides guidance on use of the MCA and includes advice on when and how to involve a consultee (ideally someone who is close to the person lacking capacity and knows them well personally, rather than a paid carer) to advise on the values and preferences of the person lacking capacity and whether they would have wished to take part in research had they been able to give consent. Whether or not a consultee is involved in the consent process, researchers should seek a person’s assent and respect any indication of their dissent to participating in research, expressed through verbal or non-verbal behaviour, and exclude those who dissent from the research [9].

Some researchers find the MCA and accompanying guidance difficult to interpret and implement (2, 10). Although the MCA’s vagueness around the meaning of capacity in relation to research allows researchers some flexibility, it also limits opportunities for a standardised process for determination of capacity and can cause researchers a great deal of unease [11, 12]. Researchers are concerned about the subjectivity of determining capacity and the tensions between adhering to legislation and the principle of non-maleficence, due to the potential to cause distress by conducting unnecessary tests or over/underestimating a person’s capacity [11, 12]. There is ambiguity around the logistics of determining capacity (e.g., measures to use and how to introduce the need for consultee involvement (11) and there are concerns about the time intensive nature of this work balanced against the demands of research timescales (10, 11).

Researchers who carry out stroke research have also reported a lack of knowledge, skills and confidence in supporting people with other capacity-affecting conditions such as stroke related aphasia (10), highlighting a lack of training, tools and time as particular barriers. Research studies including people with stroke aphasia as participants, rarely document study processes used to support their recruitment [13]. The authors [13] called for person-centred, individual tailored consent processes to address the complexity of consenting people with stroke-aphasia for research. The need to build capacity and capability within the wider dementia research workforce has been recognised [14], and programmes such as the National Institute for Health Research Three Schools Dementia Research Programme (https://www.sscr.nihr.ac.uk/dementia-research-programme/about/) have been developed to target funding in this area. However, literature on, and initiatives around upskilling dementia researchers in consent processes are lacking.

There has been some work to develop resources and guidance to support researchers in consenting people who lack capacity such as the outputs from the CONSULT study (https://www.capacityconsentresearch.com/), and training resources on broader aspects of inclusion of people with capacity and communication needs by the ASSENT team (https://www.uea.ac.uk/web/groups-and-centres/assent). A small number of tools have been developed, such as the Consent Support Tool (15) and The Evaluation to Sign Consent Tool [16]. Neither have been developed specifically for people with dementia, nor do they seek to incorporate informal person-centred approaches and minimise the use of formal testing, which can feel threatening to people with dementia [11]. There is also growing support for researchers, from organisations such as the Alzheimer’s Society, to ensure people with dementia are involved in shaping the research process from the very start, often as funded co-applicants. At the time of writing this article, a recent government response to the review of clinical trials outlined plans to accelerate dementia trial delivery [17]. Despite this, little is known about the challenges for, and training needs of researchers who are determining the capacity specifically of people with dementia to consent to research.

It is valuable to further understand the context for consent processes described in dementia research studies undertaken within England and Wales since the introduction of the MCA 2005. The National Institute for Health Research Clinical Research Network Portfolio, at the time of writing, has 383 completed studies on dementia on their database since 2008. A similar search of the Welsh database identified a further 33 studies. It was not within the scope of this paper to interrogate all of these studies, therefore a sample of 41 (10%) of these studies were randomly selected (using random.org) by the current authors to explore how consent processes were described. Of these 41 studies, 24 included people with dementia as participants, whilst others focused on caregivers or other participants. Ten of the 24 studies that included people with dementia as participants, included those who could and those who could not consent, whilst for a further seven it was unclear whether both these groups were included. Thirteen of the 24 studies that included people with dementia provided some description of a consent process (though five of these were very brief, and only 10 mentioned the MCA). Three of the 24 studies described used accessible information sheets, and one a validated questionnaire. Three of the studies described the skills of the researcher/s and the training given to support capacity assessment. This lack of information on consent processes potentially reflects underreporting, due to current journal and reporting standards not requiring this information. As a result, the opportunity for shared learning and improving process is lost.

Several authors [18–21] have, however, provided valuable work to inform researchers in supporting people with dementia to take part in research. People with dementia are often able to participate in decision-making in the mild stages of the condition, but this becomes more difficult as the disease progresses [18]. The literature on decision-making skills of people with dementia highlights specific areas of potential difficulty, such as language comprehension, and reasoning as the cognitive domains most likely to present a barrier [4]. Researchers in other legal jurisdictions have described person-centered, guiding principles and recommendations for researchers when seeking informed consent in studies involving people with dementia (e.g [19] - Ireland; [20] - India; [21] - Canada). They suggest strategies such as getting to know a person to prepare to provide appropriate support [18], simplifying consent forms, and using a visual memory aid [18, 19]. However, there is still a lack of detailed guidance and training within England and Wales on the real-life application of these principles, to help researchers gauge capacity for decision-making during recruitment e.g., advice on effective practical strategies and ‘in the moment’ communication practices (10). Appropriate knowledge of, and skills in, determining capacity are an ethical imperative if potential research participants are not to be wrongly included or excluded from research.

There is a need for evidence-informed guidance and training for dementia researchers in all areas of dementia research, in determining capacity to consent to research. This would address inequalities, by enhancing opportunities for people with dementia to participate in research informing their care and support. However, to our knowledge, whilst reflective articles have highlighted the challenges and potential facilitators in this area [11, 12], there is little understanding of the skills and needs of the researchers themselves.

## Methods

### Aims

This study aimed to understand the experiences and opinions of researchers who recruit people living with dementia to research projects, to inform the future development of training resources. We aimed to ascertain:

i. How confident and competent researchers feel about determining capacity in the context of recruiting people with dementia to research.
ii. The nature of and their views on any training and support they have experienced.
iii. Their perceptions of future training and support needs in this area and how these might be addressed.

### Design and setting

A mixed method [22], cross-sectional, electronic survey was conducted. A survey was considered the most appropriate data collection method due to the lack of existing data on this topic and therefore the need to obtain a wide range of views to understand the broad landscape. It was anticipated that using anonymous survey methods would reach researchers from across England and Wales, with a range of experience and expertise. This article has been informed by the consensus-based checklist for reporting survey studies [23].

### Ethics

The study was approved by the Chairs of UCL Language and Cognition Department Ethics on 21st November 2022, Project ID LCD-2022-11. All work undertaken in this study was conducted in accordance with the Declaration of Helsinki. Informed consent to participate was obtained from all of the participants in the study. All data were anonymised and stored securely in line with the Data Protection Act, 2018 and UK General Data Protection Regulation guidance, 2016.

### Survey development

Based on best practice guidance for survey research (American Association for Public Opinions Research: AAPOR - https://shorturl.at/azET4; [24]. we developed a prototype survey using Qualtrics (2009) software secure survey tool. This captured demographic data that would allow us to describe the respondent group (country of work, age, gender, ethnicity, professional background, length of time working in dementia research and qualification level). To address the research aims, questions were designed to ascertain confidence levels relating to determining capacity, perceived barriers and facilitators to determining capacity, previous training and what was helpful/not helpful about this, existing resources perceived as useful, potential benefits of future training and suggestions for the content of such training. We used a mix of closed questions, Likert scales [25] and open response fields to capture *‘the* “*why” that complements quantitative results, helping to tell a more nuanced story with the data’* (26: p1). To reduce participant burden, the survey was planned to take no longer than 15 min to complete.

The prototype survey was piloted by three researchers with experience of recruiting people with dementia to research projects, who were asked to comment on the design, wording (e.g., how easy the questions were to understand), ease and duration of completion and any other suggestions to improve the design. All three researchers took approximately 10 min to complete the survey. Based on their feedback several questions were refined to improve readability e.g., not to include all the response options in the question, given they are present as response options. Following refinement, the survey was published on Qualtrics. The final version is shown in Appendix One.

### Participants and recruitment

Potential participants were eligible if they were researchers with experience of recruiting people with dementia within England or Wales, where MCA legislation applies. There is a lack of population size data for these target respondents. Therefore, rather than calculating a probability or non-probability sample size, it was more appropriate to take a pragmatic census approach to recruitment, whereby responses are desired from as many participants in the undefined target population as possible [27].

The survey was advertised via social media, dementia research networks, (the National Institute for Health Research Dementia Researcher [28], recipients of NIHR Dementia Research Fellowships and Career Development Awards, and DemiQual [29]), and emails to relevant university research departments. Before completing the survey, potential participants were able to click a link to access further study information and to provide consent. The survey was open for ten weeks, to capitalise on weekly social media reminders and encouragement to respondents to share the survey in a snowballing approach [30]. Responses were considered invalid and therefore excluded from analysis if: consent questions were started/completed but survey questions not attempted, only consent and demographics questions were answered, or respondents closed their browser mid-way through completing the survey (the latter was treated as withdrawal).

### Analysis

Closed field responses were analysed using descriptive statistics. The sample did not allow for the use inferential statistics, although some tentative comparisons have been made (see results).

Open field responses were analysed using content analysis, described by Patton (31: p453). as a *‘qualitative data reduction and sense-making effort that takes a volume of qualitative material and attempts to identify core consistencies and meanings.’* Taking a more in-depth analytic approach such as reflexive thematic analysis [32] was not appropriate for data collected via online survey, where responses are typically brief, with no opportunities for researchers to probe for deeper understandings [33]. The open-ended responses were intended to enhance the quantitative fundings rather than produce standalone rich insights [34].

We followed the steps of ‘conventional content analysis’ [35]. SG and AV engaged in multiple readings of the entire data set and separately coded for initial categories derived directly from that data (inductively). Where meanings were unclear, data was left uncoded. SG and AV then compared categories and agreed how they could be merged and streamlined, thus deciding on the preliminary code set. They then recoded the data deductively and inductively, examined all data within each code, and agreed on codes that could be split, combined or abandoned. Finally, they considered how codes could be reframed into themes, to provide a coherent explanation of participants’ opinions on their confidence, competence and training needs relating to determining capacity.

## Results

There were 86 survey responses, 14 of which were excluded from the study as the consent section was not completed. Twelve were excluded from the study as participants completed the consent section but closed their browser mid-way through. 60 complete responses were included in the analysis.

### Respondent demographics

Of the 60 respondents, three (5%) reported doing research in both England and Wales, 50 (83%) respondents reported doing research in England only and seven ( 12%) respondents did not complete this field. 49 respondents were women (82%; see further demographic data in Table 1).

**Table 1:** Respondents’ demographic data

Demographic data	*n* (%)
Age 18–30 years 31–40 41–50 51–60 61+	20 (33%) 24 (40%) 5 (8%) 6 (10%) 5 (8%)
Ethnic background White Irish White British/English/Scottish/Welsh/Northern Irish *Dutch* *German* *Slav* *Italian* *European* *English/Indian* Filipino Cypriot Chinese Indian Pakistani N/A Caribbean White and Black African	5 (8%) 35 (58%) 3 (5%) 1 1 1 3 (5%) 1 1 1 1 2 2 1 1 1
Professional background: Researcher Dementia charity manager Clinical academic AHP Clinical academic medical Clinical academic nursing Other (inc. Clinical academic Health care scientist, Clinical academic – psychology, GP, Academic- sociology, Nurse Dementia carer turned dementia PhD student)	37 (62%) 1 (2%) 6 (10%) 6 (10%) 3 (5%) 7 (12%)
Highest qualification: BSc Msc PhD Not answered	2 (3%) 26 (43%) 31 (52%) 1 (2%)
Number of years recruiting people with dementia: < 1 year 1–2 years 5–10 years 2–5 years 10–15 years 15 years +	6 (10%) 10 (16%) 16 (27%) 11 (18%) 5 (8%) 3 (5%)

### Experience of determining capacity to consent

**Fig. 1:**
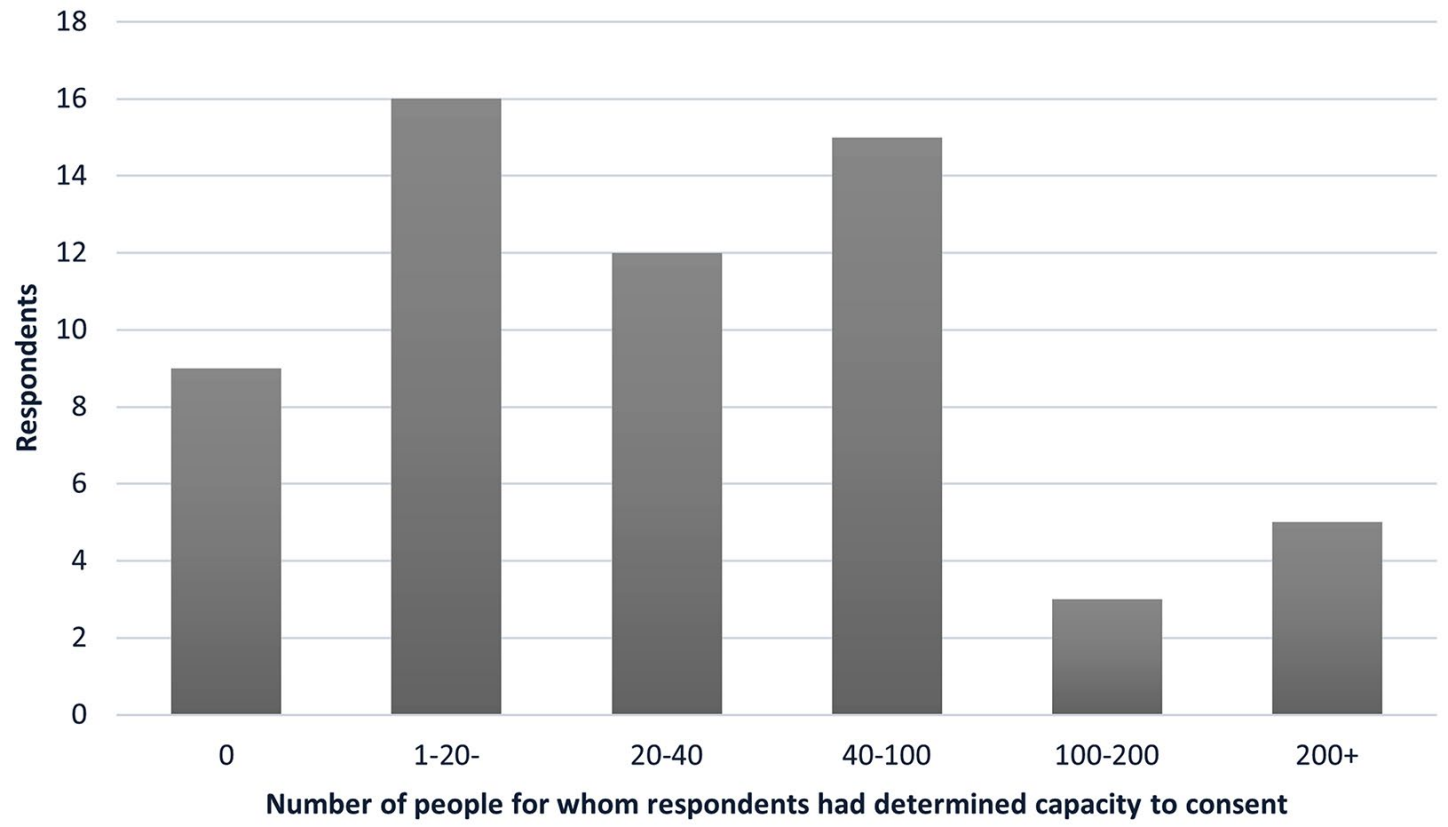
Number of people for whom respondents had determined capacity

**Fig. 2:**
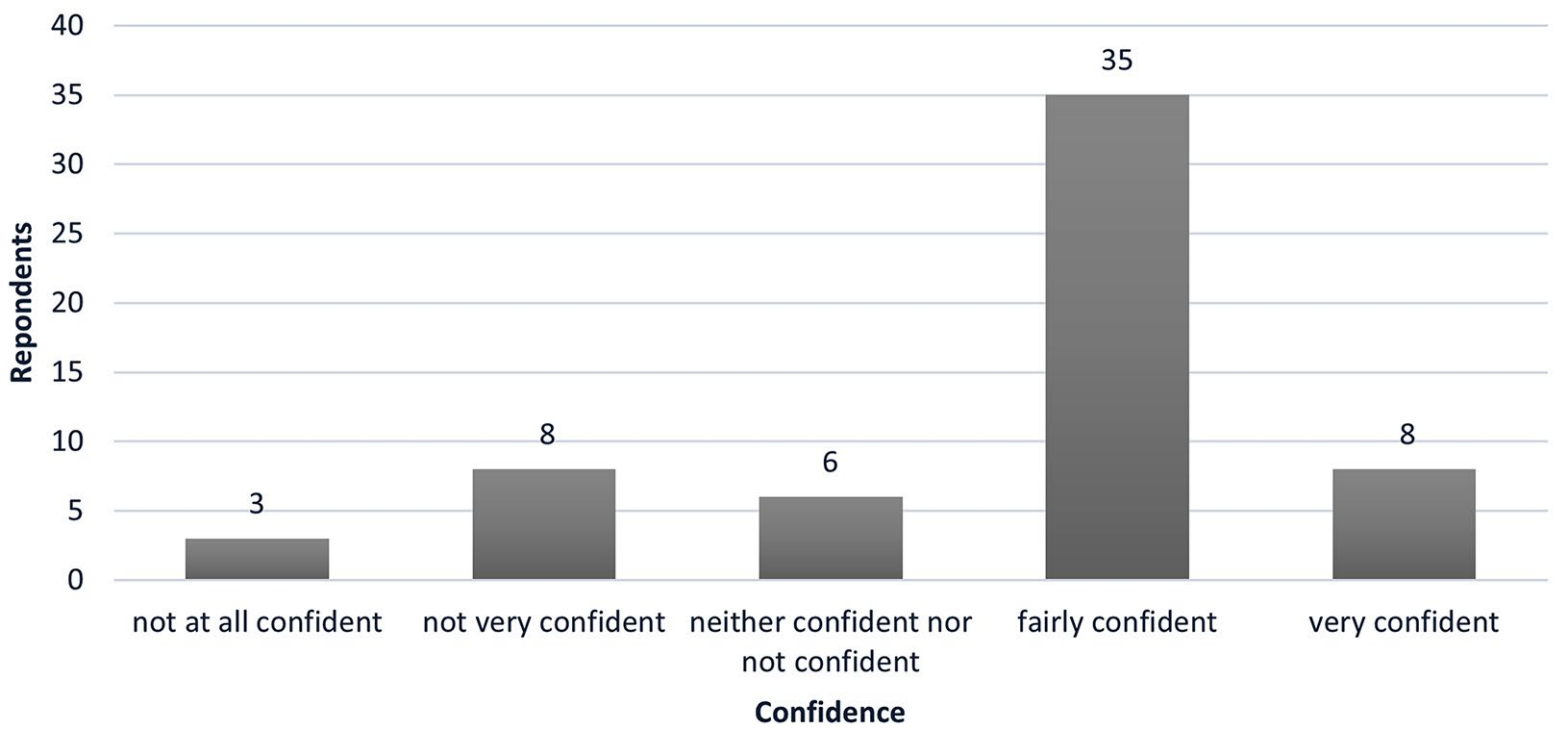
Confidence in determining capacity to consent

Nine respondents (15%) had no experience of determining a potential participant’s capacity to consent to a study (Fig. 1), whilst 43 (72%) had determined between one and 100 participants’ capacity to consent, and seven (12%) had determined more than 100 participants’ capacity to consent. Respondents with fewer years’ experience had generally recruited fewer participants. All respondents with less than one year of experience (*n* = 6) had recruited 1–20 participants, respondents with 1–2 years’ experience reported recruiting 0 (*n* = 1), 1–20 (= 3) or 20–40 participants (*n* = 4). Two participants with 1–2 years’ experience reported recruiting 40–100, or more than 200 participants respectively. Participants with 10–15 years’ experience reported recruiting 1–2 participants (*n* = 1), 40–100 (*n* = 3), 100–200 (*n* = 1) and participants with more than 15 years’ experience reported recruiting 40–100 (*n* = 1) or 100–200 (*n* = 2). Most respondents felt fairly confident (35, 58%) or very confident (8, 13%) in determining capacity to consent (Fig. 2). Six respondents were neither confident nor not confident, whilst eight (13%) were not very confident and three (5%) not at all confident. Of those who rated themselves as very confident or fairly confident, a total of eighteen had more than 15 years’ (*n* = 14) or 5–10 years’ (*n* = 4) experience in recruiting people with dementia to research, whilst ten had less than one year or 1–2 years’ experience. Similarly, of those who rated themselves as not very confident or not at all confident, one respondent had 10–15 years’ experience, and four had less than one year or 1–2 years’ experience. This suggests that experience in recruiting people with dementia to research did not necessarily mean respondents felt more confident about determining capacity.

### Training to determine capacity to consent

Two thirds of respondents (40, 66%) reported having had previous training on how to determine capacity to consent. Of these 40 respondents, 15 had clinical backgrounds (comprising 75% of the respondents with a clinical background). Just over two thirds of the entire respondent group (41, 68%) felt they would benefit from training or further training to address any uncertainties in their skills.

### Analysis of open-ended responses

Data collected from respondents who answered open questions relating to researcher confidence, competence and future training needs informed the development of six main themes: (1) **Researcher uncertainties** (*subthemes: knowledge of, and confidence in, the process, determining understanding and retention, juggling family members, and managing the fluctuating nature of capacity)* (2) **Lack of time, (3) Balancing information complexity with accessibility, (4) Gatekeepers**, **(5) Existing enablers** (*subthemes: training and background, tools and resources, communication strategies*), **and (6) Envisioning future training** (*subthemes: experiences of past training and future training needs).* We now present these themes, illustrated with quotes from the online data where responses were expansive. The presented quotes are not linked to respondent characteristics, as this would threaten anonymisation, due to low numbers within some demographic categories.

### 1. Researcher uncertainties

Whilst a limited number of respondents reported having no uncertainties, the majority articulated their worries about how to go about aspects of determining capacity.

#### Knowledge of, and confidence, in the process

Respondents expressed uncertainties about who should determine capacity and where to start:

‘*Who is responsible for judging capacity?’*

‘*…not sure on what questions to ask and how.’*

‘*I don’t know all the steps I need to follow to determine if someone has capacity. I don’t know if there are any steps even.’*

There was also uncertainty around how formal or informal the process should be:

‘*Knowing whether something formal needs to be in place or if it is sufficient to assume capacity unless there appears to be reason to be concerned.’*

There was a sense of not knowing what is not known:

‘*I’m confident that I am able to follow ethical processes but am sometimes concerned there are legal processes that I am not aware of.’*

Respondents expressed uncertainty over how to justify decisions:

‘*…making sure I have justifiable reasons for why I believe someone does, or does not, have capacity.’*

This leads to a lack of confidence, worries about making mistakes, and fears that mistakes could have a negative emotional impact on people with dementia. For example, one participant expressed a ‘*fear of getting it wrong’* and another described worries about *‘inadvertently causing distress if judgment of capacity is incorrect.’*

These fears are fuelled by the knowledge that capacity judgements are subjective; there will always be doubt and no way of checking:

‘*Can’t always confirm. Some degree of doubt is present.’*

#### Determining understanding and retention

Whilst researchers were aware of the need to tailor research information to a person’s level of comprehension, they had uncertainties about how to determine whether the recipient has understood and retained enough information to make an informed decision regarding participation (MCA, 2005). For instance, speaking about people with dementia being recruited to studies, one respondent stated that it is:

‘*…sometimes difficult to ascertain if they understand the entire study if it is complex…difficult to tell…if they are quiet or give short answers.’*

Respondents expressed concerns about the impact of power imbalance or social dynamics on determining understanding:

‘*I’m worried that someone with dementia might say they understand something when they don’t just out of social obligation or because they’re embarrassed.’*

These uncertainties are amplified when the person with dementia gives brief answers to questions, is nervous, is adept at hiding their comprehension difficulties or has unmanaged hearing loss.

‘*Some people have very good masking/compensating mechanisms…excellent conversationalists.’*

#### Juggling family members

Respondents were uncertain how to deal with situations where family members talk over or answer questions on behalf of people with dementia:

‘*It can be difficult to assess someone’s capacity when there is a family member answering questions for them.’*

There may also be differing desires to participate in the research between the family members, and the person with dementia which can influence the dynamic, when carers are supporting communication:

‘*Those around the person being keen for them to take part, so interjecting in the assessment with their views and giving answers for the person.’*

There can also be differing views on capacity within the family, which respondents felt could influence capacity decisions. At times, family members were said to:

*‘…not want to accept that the person with dementia may not have capacity’* or

‘*…present biased views on [the person’s] capacity, which could all blur my clarity on the person’s capacity to give consent.’*

Another respondent raised uncertainties around how to involve family carers in a way that does not cause the person with dementia to become embarrassed and/or disempowered:

‘*It’s difficult to include carers in the process without making the person with dementia feel embarrassed or that I’m implicitly implying that they lack capacity. But including a carer would be very helpful, so this is difficult to navigate*.’

#### Managing the fluctuating nature of capacity

Respondents were particularly concerned about how to take account of day-to-day fluctuations in cognition associated with dementia. In one respondent’s words there were uncertainties around *‘how to incorporate ongoing capacity judgement throughout a study lifetime.’*

There was also a worry that fluctuations in cognition might lead to unnecessary exclusion of people with dementia in research:

‘*My uncertainties are around fluctuating cognitive abilities…and the fact that capacity to consent in the here and now might not be there even after a short period of time. Or in the reverse circumstance, that I exclude someone from research based on their incapacity to give consent at a given time, when potentially shortly after they could be involved in research.’*

### 2. Lack of time

Respondents felt strongly that determining capacity requires the development of trusting relationships over time, with researchers collecting background information (e.g., about how the person usually demonstrates a decision) and getting to know the person with dementia:

‘*When you don’t have much time to get to know a person prior to the start of the research, it can be more challenging to assess changes in capacity during the data capture.’*

However, lack of time was commonly cited as a barrier to conducting such person-centred capacity judgements:

*‘Only short amounts of time with the individual, over phone or zoom, is very hard*.’

Respondents described trying to do this work whilst responding to the pressure of competing research priorities such as recruitment targets and tight timelines, resulting in *‘time constraints on [participant] visits.’* Respondents identified a perceived *‘…conflict between recruitment targets and doing an objective assessment of capacity.’*

### 3. Balancing information complexity with accessibility

Respondents identified a conflict between ethical requirements to explain specific concepts such as study procedures, informed consent, anonymity and data protection on the one hand, and the communication needs of people with dementia:

*‘The complexities of data protection are often poorly described in [study information] and consent forms and it is bewildering and can be difficult to explain the meaning.’*

Presenting abstract concepts in accessible ways was seen as challenging, especially for complex research studies with several elements. Even respondents with experience of clinical capacity assessment and developing accessible resources, e.g., speech and language therapists, found that this experience was not necessarily transferable to the research context, and the need to balance ethical requirements with participants’ need for accessible research-specific information:

*‘As someone with a clinical background, I have some experience of assessing capacity. However, formal assessment for capacity to consent to collection and, more importantly, use of data, I am less confident with. I am concerned that consenting […] might be too abstract a concept/process for some people with reduced/altered/changing cognition.’*

### 4. Gatekeepers

Family members and formal carers were sometimes seen to function as gatekeepers to research participation, making decisions about whether person with dementia would be suitable for or would want to take part in the research, thus preventing researchers from even getting to the stage of determining capacity:

‘*Clinical staff and sometimes families gatekeep and don’t even let you talk with the person with dementia.’*

Experiences of gatekeeping extended to regulatory bodies, seen to block researchers from engaging people with dementia in the process of establishing capacity to consent:

‘*…the ‘blanket’ label of vulnerable is applied to people living with dementia and is embedded in key processes such as seeking ethics approvals…it was suggested to me by an Ethics/REC some years ago that the care team should assess capacity rather than the researcher - this felt undermining.’*

### 5. Existing enablers

Respondents identified existing sources of support, skills and personal attributes that they felt were enabling their capacity judgment work. These included their professional training and background, tools and resources, and communication strategies (sub-themes).

#### Training and background

Some respondents felt that formal training, such as that delivered by the National Institute for Health Research (https://www.nihr.ac.uk/health-and-care-professionals/training/good-clinical-practice.htm) and https://cpduk.co.uk/courses/nihr-clinical-research-network-informed-consent-with-adults-lacking-capacity), had supported them to develop skills to involve people who may lack capacity in research. Other helpful sources of support included experiential learning, particularly talking to and observing more experienced researchers. Respondents also felt that having a secure and detailed understanding of the research study allowed them to feel more confident, as did having a clinical background, although as identified in theme 3 this was not always the case.

#### Tools and resources

Whilst resources to support capacity judgment were felt to be generally lacking, available tools which respondents reported as helpful were the MCA code of practice, the ‘capacity and consent to research resources’ website (https://www.capacityconsentresearch.com/), homegrown checklists to guide determination of capacity and consent based on the four stages of the MCA, the ‘Dementia Enquirers Gold Standards for ethical research [36],’ Talking Mats [37] and research papers on this topic (e.g., 11). Respondents also explained that working with patient and public involvement and engagement (PPIE) contributors throughout a research project was a valuable resource, offering support and advice. PPIE involves people who have lived experience of an area of health or social care informing and shaping research and its dissemination in that area.

#### Communication strategies

Respondents described several communication strategies they felt they could use in supporting potential participants to consent including taking the time, wherever possible, to build relationships and trust with potential participants (although Theme 2 explores the barrier created by time constraints) and bringing an *‘openness’* and *‘willingness to engage in everyday informal conversation.’* Asking family members for advice on communication strategies specific to the individual, developing accessible Participant Information Sheets and consent documents, ensuring that there is a private space for consent conversations and having those conversations *‘over a cup of tea,*’ were also strategies cited as being within respondents’ existing ‘toolkits.’ In conversations, respondents identified several strategies including *‘providing a summary of information, and breaking things down into smaller chunks*.’ They also described presenting graded information (the simplest messages first, becoming more complex to gauge understanding), checking comprehension by asking people what they have understood and/or inviting their questions about the research.

### 6. Envisioning future training

Where respondents had experienced some training in this area, they regarded the practical elements as most helpful e.g., shadowing and debriefing with colleagues. A lack of focus specifically on the challenges relating to dementia (as opposed to other conditions where capacity is relevant), was felt to be less helpful:

*‘Some information seemed very detached from the reality of working with someone with dementia*.’

They reported a tension between courses focusing on ensuring trainees understood the legal aspects (the MCA and its background) rather than the *how* of determining capacity. In respondents’ experience, training encountered had not provided sufficient in-depth and nuanced consideration of specific challenges and complexities related to involving people with dementia in research:

‘*It’s not all black and white, yes/no.’*

Respondents overwhelmingly wished for future training in this area to be practical in nature. Table 2 summarises specific suggestions from respondents for future training in this area.

**Table 2:** Respondents’ suggestions for future training

Suggestions for future training in determining capacity
· Involvement of people with lived experience in designing and running the training
· Discussion of real-life scenarios, specific to dementia, and the complexities of determining capacity in this population, including how to involve carers
· Determining capacity in diverse populations
· Exploration of communication strategies (e.g., how to communicate complex ideas, balance differing opinions on capacity and judge comprehension)
· Practising skills through activities such as role play, quizzes and online tests
· Discussion on concepts such as the rights and autonomy of people with dementia and power imbalances *(e.g., ‘I think training should be much more reflective and less directive/prescriptive to genuinely engage with the debates around consent.’*)
· Support beyond the course, through shadowing and mentoring
· Refresher courses

## Discussion

The findings from this study add to current research evidence on the experiences and opinions of researchers working with people who may lack capacity, specifically with people with dementia, an area as yet not explored in great detail. Similar to previous research in stroke aphasia (10), this survey study of English and Welsh researchers demonstrates that despite having experience in, and education on, the legislative aspects of capacity assessment, their levels of confidence in determining a person with dementia’s ability to consent to research were not consistently high. Their experiences of training highlighted a lack of training specific to working with people with dementia and their families, and a need for practical training to support them to assess a person’s ability to understand and weigh up information. Respondents emphasised a need for time, resources and tools to enable them to get to know a person with dementia and their communication needs, in order to provide appropriate supports in the decision-making process. They flagged the tensions in their role, and the pressure to recruit participants. Concerns expressed about clinical staff and regulatory bodies gatekeeping access to people with dementia, making decisions about their suitability for research involvement, is contrary to the MCA, which requires the decision-maker (researcher) to determine capacity, albeit alongside others with in-depth knowledge of the person. This indicates that there is confusion about implementing the MCA amongst not just researchers, but those with whom they need to collaborate. Data collected from respondents addressed the key objective of the study, by highlighting a need for specific training and guidance on how to overcome such challenges and how best to include people with dementia in research. Interestingly, uncertainly over recording the processes by which capacity is determined, and documenting the outcome, were not seen as strong themes, however future research could seek to explore this area.

This study builds on the current and contemporary literature in this field. Our findings fit with existing knowledge that researchers are uncertain about legal frameworks governing research with adults lacking capacity more broadly (not just regarding how to determine capacity) [38]. Lack of confidence about operationalising the MCA and of access to training have also been found amongst health care professionals (HCPs) carrying out capacity assessments in clinical settings [39]. Our respondents’ fears of getting it wrong resonate with the concerns of researchers and health care professionals about conducting trials involving people who lack capacity, that *‘the ethics police will come for you.’* (40: p7). Like our respondents, HCPs wish for training to focus on practical issues [41]. Combined with this growing evidence, our findings make a clear case for a more joined up approach to research on the processes that embody the legislative components of assessing of decision-making capacity.

Understanding the needs of researchers undertaking capacity assessments will better inform the development of future guidance and training. This builds on previous work in this field demonstrating that researchers working with people with stroke-related aphasia do not feel confident in making judgments about people’s ability to consent (10). There is guidance for researchers, not least the MCA and accompanying Code of Practice, but also more specifically resources such as the capacity and consent to research resources’ website arising from the CONSULT study and resources developed by the ASSENT team. Whilst these resources do try to take account of complex situations, and cognitive and communication difficulties, they were not specifically designed for researchers working with people with dementia. This study extends previous reflective work [11, 12] by identifying the specific and current needs of researchers working with people with dementia across England and Wales. There is great potential to contribute to a developing suite of researcher resources, with specialized training tools on determining capacity for research related to dementia.

People with dementia have unique support needs when being consented for participation in research. Future research to interrogate the reporting of consent procedures in research with people with dementia across England and Wales since the publication of the MCA (2005) would inform our understanding of current practice. Given current significant advances in the field of dementia research, potential participants are often making consent decisions about becoming involved in a variety of research, from small scale theory development projects to multicentre, multistage intervention trials, including a range of qualitative, quantitative and mixed methods. The need for inclusive consent processes applies across all these types of research. Survey respondents highlighted that people with dementia may need to understand a large amount of complex information in relation to their potential participation. They and their families may have personal agendas about participation, for instance where studies are trialling potential life changing, possibly curative interventions. Yet people with dementia may present with fluctuating capacity to consent over the course of a study, which respondents emphasised as being difficult for them to manage both practically and ethically. Dewing [42] describes the concept of process consent, providing principles to consider when supporting older people, including spending time getting to know them. Importantly, Dewing provides several examples of how to interpret what the person with dementia says, yet does not describe what the researcher should ask to check they understand information provided. Given researchers identified time as a barrier, it would be useful to explore how Dewing’s description of process consent could be modified in more time critical research. It seems timely that a set of reporting standards, guiding the appropriate enactment of the MCA, should be developed and implemented by peer reviewed journals to ensure transparency. Given the likely increase in the number and complexity of future trials in this field, evidenced by the recent UK government’s plans to pilot a new clinical trial delivery accelerator for dementia research [17], there is an urgent need for such reporting standards.

Survey respondents highlighted that care partners can be helpful in supporting communication between the researcher and the person with dementia during consent conversations. However, they also identified concerns about wanting to employ strategies that do not disempower the person living with dementia during these triadic interactions. Conversation Analysis (CA) is a method for examining the turn-by-turn detail of talk-in-interaction [43]. Using CA, several studies developed training to support health and social care professionals [44] or care partners [45] in interacting with people with dementia in a multimodal way that foregrounds relationships and power dynamics. These training programmes used CA to explore moment-by-moment phenomena within conversations and identify behaviours acting as barriers and facilitators to interactions. It seems logical that CA could provide an ecologically valuable method of understanding what researchers say and do that assist or hinder conversations around consent. Indeed, Wade et al. [46] used CA methods to understand how researchers can maintain equipoise during consent sessions, presenting information in an unbiased way. Similarly, future research using CA methods to explore interactions during consent sessions with people with dementia could inform methods for accessible presentation of information.

### Limitations

Respondents in this study were recruited via online social media sites, and research networks known to the authors. This may have contributed to the gender bias in the respondent sample. Obtaining a representative sample in future research will be helpful to ensure the data collected is representative of the research community, and asking about whether respondents have undertaken clinical trials and other types of research would be useful. Additionally, whilst this study surveyed the views of researchers, future research in this field should ensure that the views of people with dementia and their care partners are considered. Any future training intervention must be situated in the needs of the people with dementia and their care partners and co-produced with them.

Despite the eligibility criteria outlined at the start of the survey, indicating respondents were eligible for participation only if they undertook research in England and Wales, seven respondents did not complete the field indicating whether they did research in England and/or Wales. A limitation of the survey design was that it was not compulsory for respondents to complete this question.

Survey studies are a useful method to reach a large number of respondents, however, the nature of the study design limited the qualitative data collected in that it is not possible to probe or clarify responses. Indeed, it might have been useful to explore the underlying meaning of several responses. Despite this, there was a large volume of qualitative data collected. In using a content analysis approach to identifying themes in the data the researchers themselves acknowledge their positions as both clinically trained speech and language therapists and dementia researchers may have biased their interpretation of the data. However, this position also enabled them to identify specific concerns voiced by researchers that might be addressed through the development of CA informed training.

This research has a particular focus on researchers within the legal jurisdiction of the MCA 2005: England and Wales. However, whilst the geographical legislative boundaries may mean subtle or larger differences in legal practice, many principles relating to supporting people in decision making are universal as advocated by the United Nations Convention of the Rights of People with Disabilities (https://www.un.org/disabilities/documents/convention/convoptprot-e.pdf). Consequently, the recommendations for researchers working with people with dementia outlined in this paper may be considered good practice at an international level.

## Conclusions

This survey study of English and Welsh researchers demonstrates that despite having experience in, and education on, the legislative aspects of capacity assessment, they identified training needs in undertaking conversations around consent with people with dementia, who may have fluctuating capacity. Despite support from care givers, researchers have little practical guidance on methods of determining a person’s ability to understand or appreciate the information they have provided during the consent process. Given the development of large complex trials within dementia research, there is an urgency to develop specific and practical guidance and training for researchers working with people with dementia and their families.

### Electronic supplementary material

Below is the link to the electronic supplementary material.

Supplementary Material 1

## Data Availability

The deidentified data that support the findings of this study were collected through and are held by University College London. Quantitative data is available from the authors upon reasonable request to the first author: s.a.griffiths@ucl.ac.uk. Qualitative data will not be available, to protect participant anonymity. Requests to use data will be submitted on a standard form and reviewed by the authors prior to data-sharing agreements being developed.

## Abbreviations

CA: Conversation Analysis
HCP: Healthcare professional
MCA: Mental Capacity Act
