# Supplementary material for: Determining capacity of people with dementia to take part in research: an electronic survey study of researcher confidence, competence and training needs
Source: BMC Med Ethics. 2024 May 28;25:65. doi: 10.1186/s12910-024-01056-6 (PMC11131177; doi:10.1186/s12910-024-01056-6)
Supplement: Supplementary file 1 — Supplementary Material 1 [file 12910_2024_1056_MOESM1_ESM.docx]

**APPENDIX ONE**

**SURVEY QUESTIONS:** Determining capacity of people living with dementia to take part in research: an electronic survey study of researcher confidence, competence and training needs.

Q1 What country do you do research in?

- England
- Wales
- England and Wales
- Only outside of England and Wales

Q2 How old are you?

- 18-30
- 31-40
- 41-50
- 51-60
- 61+

Q3 What is your gender?

- Male
- Female
- Non-binary / third gender
- Prefer not to say

Q4 What is your ethnic background?

- White British/English/Scottish/Welsh/Northern Irish
- White Irish
- White Gypsy or Irish Traveller
- Any other white background (please describe) _______________________________________________
- White and Black Caribbean
- White and Black African
- White and South Asian
- Any other mixed / multiple ethnic background (please describe) __________________________________________________
- British Asian
- Indian
- Pakistani
- Bangladeshi
- Chinese
- Any other Asian background (please describe) __________________________________________________
- Black British
- African
- Caribbean
- Any other Black/African/Caribbean background (please describe) _________________________________________________
- Arab
- Any other ethnic group (please describe) _________________________________________________

Q5 What is your professional background?

- Researcher
- Clinical Academic - Nursing
- Clinical Academic- Medical
- Clinical Academic- Allied Health Professional
- Clinical Academic- Psychology
- Other (please describe)

__________________________________________________

Q6 How long have you been involved in recruiting people with dementia to participate in research?

- I have never recruited anyone with dementia to a research study
- <1 year
- 1-2 years
- 2-5 years
- 5-10 years
- 10-15 years
- 15 years +

Q7 What is the highest level of qualification do you have?

- BSc
- MSc
- PhD

Q8 How many people with dementia (rough estimate) have you determined capacity for over the course of your research career?

- 0
- 1-20
- 20-40
- 40-100
- 100-200
- 200+

Q9 How confident do you feel about making judgements (being a decision maker) on a person's capacity to consent?

- 1 Not at all confident
- 2 Not very confident
- 3 Neither confident nor not confident
- 4 Fairly confident
- 5 Very confident

Q10 What are your uncertainties, if any, around determining capacity and consenting a person with dementia to participate in research?

Q12 What do you perceive as barriers to assessing capacity to consent?

Q13 What has helped you to assess capacity to consent?

Q14 Have you had any training in this area?

- Yes
- No

Q15 if yes, did the training cover (tick all that apply):

- MCA legislative Information / Updates
- Development of consent forms and participant information sheets
- Peer learning
- Practical strategies to support communication.
- Shadowing and mentoring
- Involvement of consultees
- Other- see next question.

Q16 What was helpful about the training you received?

Q17 What was not helpful about the training you received?

Q18 What tools or resources have you found helpful?

Q19 Do you think you would benefit from further training to address any uncertainties in your skills around determining capacity and obtaining consent?

- Yes
- Unsure
- No

Q20 What would you want training on this topic to focus on?

Q21 What training do you think should be available for researchers having to undertake judgements of capacity and obtaining consent?

**ENDIX ONE**

SURVEY QUESTIONS: Developing skills in dementia researchers for determining capacity and obtaining consent.

Q1 What country do you do research in?

- England
- Wales
- England and Wales
- Only outside of England and Wales

Q2 How old are you?

- 18-30
- 31-40
- 41-50
- 51-60
- 61+

Q3 What is your gender?

- Male
- Female
- Non-binary / third gender
- Prefer not to say

Q4 What is your ethnic background?

- White British/English/Scottish/Welsh/Northern Irish
- White Irish
- White Gypsy or Irish Traveller
- Any other white background (please describe) _______________________________________________
- White and Black Caribbean
- White and Black African
- White and South Asian
- Any other mixed / multiple ethnic background (please describe) __________________________________________________
- British Asian
- Indian
- Pakistani
- Bangladeshi
- Chinese
- Any other Asian background (please describe) __________________________________________________
- Black British
- African
- Caribbean
- Any other Black/African/Caribbean background (please describe) _________________________________________________
- Arab
- Any other ethnic group (please describe) _________________________________________________

Q5 What is your professional background?

- Researcher
- Clinical Academic - Nursing
- Clinical Academic- Medical
- Clinical Academic- Allied Health Professional
- Clinical Academic- Psychology
- Other (please describe)

__________________________________________________

Q6 How long have you been involved in recruiting people with dementia to participate in research?

- I have never recruited anyone with dementia to a research study
- <1 year
- 1-2 years
- 2-5 years
- 5-10 years
- 10-15 years
- 15 years +

Q7 What is the highest level of qualification do you have?

- BSc
- MSc
- PhD

Q8 How many people with dementia (rough estimate) have you determined capacity for over the course of your research career?

- 0
- 1-20
- 20-40
- 40-100
- 100-200
- 200+

Q9 How confident do you feel about making judgements (being a decision maker) on a person's capacity to consent?

- 1 Not at all confident
- 2 Not very confident
- 3 Neither confident nor not confident
- 4 Fairly confident
- 5 Very confident

Q10 What are your uncertainties, if any, around determining capacity and consenting a person with dementia to participate in research?

Q12 What do you perceive as barriers to assessing capacity to consent?

Q13 What has helped you to assess capacity to consent?

Q14 Have you had any training in this area?

- Yes
- No

Q15 if yes, did the training cover (tick all that apply):

- MCA legislative Information / Updates
- Development of consent forms and participant information sheets
- Peer learning
- Practical strategies to support communication.
- Shadowing and mentoring
- Involvement of consultees
- Other- see next question.

Q16 What was helpful about the training you received?

Q17 What was not helpful about the training you received?

Q18 What tools or resources have you found helpful?

Q19 Do you think you would benefit from further training to address any uncertainties in your skills around determining capacity and obtaining consent?

- Yes
- Unsure
- No

Q20 What would you want training on this topic to focus on?

Q21 What training do you think should be available for researchers having to undertake judgements of capacity and obtaining consent?
